# Supplementary material for: Predicting visual function by interpreting a neuronal wiring diagram
Source: Nature. 2024 Oct 2;634(8032):113–23. doi: 10.1038/s41586-024-07953-5 (PMC11446822; doi:10.1038/s41586-024-07953-5)
Supplement: Supplementary file 6 — CRF and ERF predictions for individual TmY4 and TmY9 cells. Analogous to Supplementary Data 3, but for TmY target types. Shown are the top four monosynaptic pathways, the strongest pathway passing through each of the top ten intermediary types (ranking from Extended Data Fig. 7), and the trisynaptic pathway Tm1–TmY–Dm3–TmY (see the section entitled Prediction of spatial normalization). [file 41586_2024_7953_MOESM6_ESM.zip › DataS4/TmY9q/720575940632913100.pdf]

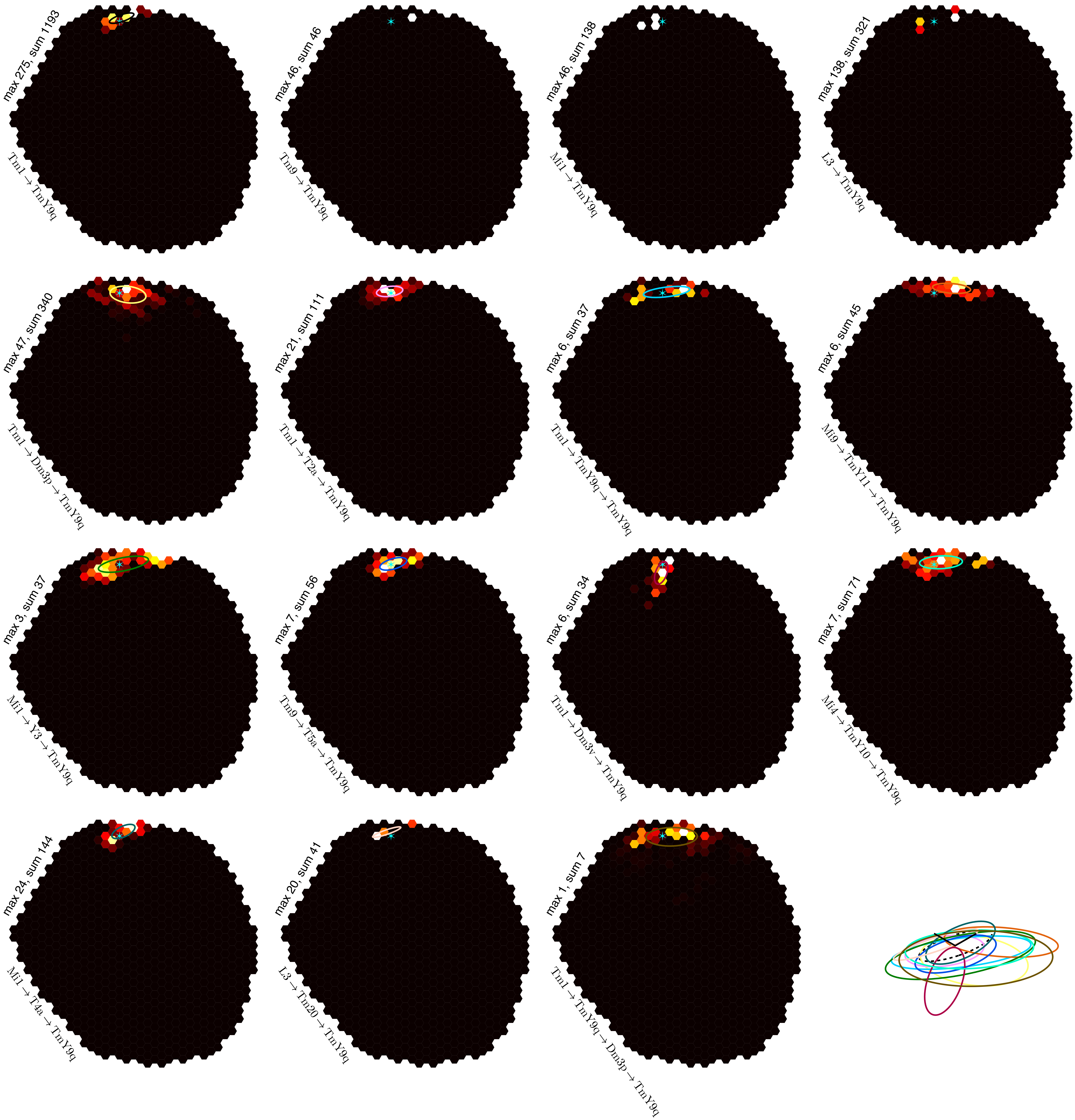
max 275, sum 1193
Tm1 → TmY9q
max 46, sum 46
Tm9 → TmY9q
max 46, sum 138
Mi1 → TmY9q
max 138, sum 321
L3 → TmY9q
max 47, sum 340
Tm1 → Dm3p → TmY9q
max 21, sum 111
Tm1 → T2a → TmY9q
max 6, sum 37
Tm1 → TmY9q → TmY9q
max 6, sum 45
Mi9 → TmY11 → TmY9q
max 3, sum 37
Mi1 → Y3 → TmY9q
max 7, sum 56
Tm9 → T5a → TmY9q
max 6, sum 34
Tm1 → Dm3v → TmY9q
max 7, sum 71
Mi4 → TmY10 → TmY9q
max 24, sum 144
Mi1 → T4a → TmY9q
max 20, sum 41
L3 → Tm20 → TmY9q
max 1, sum 7
Tm1 → TmY9q → Dm3p → TmY9q
